# Validation of D-SCOPE Questionnaire: Dietitians’ Survey of Comfort, Opinions, and Perceptions on Education in Supplements

**DOI:** 10.3390/nu17152451

**Published:** 2025-07-28

**Authors:** Margaret Harris, Keston Lindsay, Lauryn Bille, Nicole Fioretti, Andrea Hutchins

**Affiliations:** 1 Department of Human Physiology and Nutrition, University of Colorado Colorado Springs, Colorado Springs, CO 80918, USA; lauryn.bille@outlook.com (L.B.); nikki.fioretti@nationals.com (N.F.); ahutchin@uccs.edu (A.H.); 2 Athletics, Sports Nutrition Department, Southern Methodist University, Dallas, TX 75275, USA; 3 Washington Nationals Minor League, West Palm Beach, FL 33407, USA

**Keywords:** competence, attitudes, survey, perceived knowledge, dietary supplements

## Abstract

**Background/Objectives:** The field of dietary supplements is changing and evolving quickly. Registered Dietitian Nutritionists are recognized as experts in nutrition and familiarity with the usage of dietary supplements is expected. However, education on the use of dietary supplements is not equal across accredited dietetic education programs, which can lead to disparities in dietitians’ feelings of preparedness, attitudes, and consequently experience of comfort regarding dietary supplements. The purpose of this study was to create the D-SCOPE Questionnaire (Dietitians’ Survey of Comfort, Opinions, and Preparedness in Education in Supplements) and validate it. This questionnaire assesses Registered Dietitian Nutritionists’ feelings of preparedness, comfort with use, and general attitudes in the field of dietary supplements. **Methods:** Face and content validity was established with dietitian, nutritionist, and statistician input. For recruitment, 2000 national randomly selected emails were obtained from the Commission on Dietetic Registration. Registered Dietitian Nutritionists (n = 248) responded to the survey email request. Descriptive statistics (reported as means ± standard deviation), principal axis factoring (exploratory factor analysis) with a direct oblimin rotation and Cronbach’s a reliability analysis were used for validation techniques. **Results:** Five factors were created, which explained about 63% of the variance in the questionnaire. The questionnaire was generally reliable, but the factor structure could change with a non-US population. **Conclusions:** As a unit, the D-SCOPE Questionnaire shows validity and reliability in assessing Registered Dietitian Nutritionists’ perceptions of preparedness and attitudes in the area related to dietary supplements.

## 1. Introduction

The dietary supplement industry has been growing steadily over the last several decades [1]. As science and healthcare continue to advance, so do dietary supplements. More bioavailable formulations are being developed to address nutrient deficiencies. For example, iron bisglycinate is more bioavailable than iron sulfate with fewer unpleasant side effects [2]. Liposomal technology and peptides are also cutting-edge technologies helping with better absorption [3,4]. New types of products are being developed such as probiotics, vegan proteins, and collagen peptides. Consequently, questions continue to arise from patients regarding the field of dietary supplements, but the field is rife with controversies ranging from how to use these supplements to whether a patient should use them for benefits or refrain from use due to potential harm.

Registered Dietitians/Registered Dietitian Nutritionists (RDs/RDNs) are healthcare experts in nutrition and dietary supplements [5]. Through accredited training, all RDs/RDNs should have sufficient knowledge about dietary supplements to answer patient questions and use them in practice when nutrient deficiencies arise. However, new fields, such as nutrigenomics and the gut microbiome, have emerged, raising the awareness that genes and microbes can impact how nutrients are utilized and absorbed [6]. This adds a level of complexity to the field of dietetics. Additionally, the population’s continued lack of adherence to a healthy diet (particularly by the United States population) [7] indicates that RDs/RDNs, now more than ever, need to be well-versed in dietary supplement literacy, but curricula may be lacking in sufficient training.

The research in this area is sparse. Of the few United States-based studies published on this topic [8,9,10,11,12,13], most have addressed RDs/RDNs’ use or perceptions of dietary supplements, but none have addressed RDs/RDNs’ perceptions of feeling prepared to discuss and recommend dietary supplements. The last known national general dietary supplement assessment of RDs/RDNs in the United States occurred in 2012 [8]. Since then, new types of products have been developed that improve bioavailability, and new fields have emerged that bring complexity to this field, requiring more knowledge to counsel patients successfully. The purpose of this research was to develop and validate a questionnaire assessing RDs/RDNs’ feelings of preparedness, comfort with use, and general attitudes in the field of dietary supplements when practicing in the United States.

## 2. Materials and Methods

This study was approved by the Institutional Review Board of the University of Colorado Colorado Springs (UCCS Protocol 2022–090).

### 2.1. Face and Content Validity

The questionnaire was created by a research team consisting of three faculty members at the University of Colorado Colorado Springs (one statistician and two nutrition faculty members with expertise in nutrition and dietary supplements, one being an RD/RDN with over thirty years of experience in clinical practice) in 2021. In addition, four practicing RDs/RDNs in school for their master’s degree in the Sport Nutrition field contributed to the development of the questionnaire.

Eighteen questions regarding dietary supplements were developed with the intention to capture the feelings of preparedness (such as questions regarding scope of practice, ability to research, and opinions around training), comfort with use (such as questions regarding being able to distinguish between different formulations of nutrients or how to prepare herbals for therapeutic purposes), and general attitudes, which could also capture biases (such as questions regarding whether supplements were beneficial or harmful). The definition of “dietary supplements” provided in the questionnaire included the following: “Any ingredient intended to be added to the diet in any form (for example: teas, capsules, tablets, powders, tinctures) and include (but not limited to) vitamins, minerals, herbals, probiotics, prebiotics, enzymes, amino acids, fats (for example: MCT oil, borage oil, fish oil), glandular extracts, sport supplements (for example: creatine, whey protein), superfood extracts (for example: green powders, phytochemicals like quercetin, resveratrol).” Answers were rated as 1 = strongly disagree; 2 = disagree; 3 = neutral; 4 = agree; or 5 = strongly agree (DOCS1).

Once developed, ten practicing RDs/RDNs outside of the university with a range of experience from 1 year to over 30 years of practice were recruited to qualitatively evaluate the content and format of the questionnaire (face and content validity). This part of the validation process is key to minimizing bias. The edits were considered and made by the research team.

The Commission on Dietetic Registration (CDR) was contacted to obtain a list of member names from the United States. Further suggestions for edits in the questionnaire were provided by the CDR (final questionnaire is available in the Supplementary Materials online). The CDR then sent an email to a randomly selected pool of 2000 members. Emails were distributed using Qualtrics (Provo, UT). This cross-sectional survey was completed between the years 2022 and 2023. The questionnaire responses were anonymous; however a link, separated from the responses to the questionnaire, was provided for willing participants to enter their name into a draw for a gift card.

### 2.2. Construct Validity

Data were analyzed using SPSS version 29 (Armonk, NY, USA). There were 248 responses, and after cleaning, 222 subjects’ data were included in the dataset for analysis. Exclusion criteria included the following: did not consent “yes” (n = 1), not an RD/RDN (n = 2), and missing answers for the validation questions (n = 23).

There is no real consensus for sample size for factor analysis. Generally, 200 people or more is better as correlation coefficients tend to be stable at this sample size. We chose to obtain as many participants as possible, but we were only able to obtain 222 for the final analysis. This sample size falls in line with several recommendations: (1) 1/10 times as many participants as variables [14], and (2) we report an overall Kaiser–Meyer–Olkin measure of sampling adequacy ≥ 0.70 (0.80; *p* < 0.05) in the Results Section [15].

Descriptive statistics were obtained for all items. Exploratory factor analysis (principal axis factoring) with a direct oblimin rotation was used to analyze the data. This is an oblique rotation, chosen when the items are expected to be related [16]. Bartlett’s Test of Sphericity showed the rejection of the null hypothesis of an identity correlation matrix [χ^2^ (*df* = 153) = 1300.7, *p* < 0.001], suggesting that these data were suited to be factor-analyzed. Factor analysis is a commonly used statistical method for test development and scoring [16].

During the preparation of this manuscript, the authors used Chat GPT (GPT-4.0 [Large Language Model; Open AI; https://chat.openai.com/chat accessed on 24 July 2025) to create a title and acronym for the questionnaire used in this study (see additional information in the Supplementary Materials).

## 3. Results

The demographic data of the dietitians surveyed is presented in Table 1. Briefly, the average age of responding dietitians was 45.5 ± 13.7 with the average number of years working as a dietitian being 17.2 ± 12.5; the majority were female with over half earning a master’s degree. Item means and standard deviations are presented in Table 2.

All items possessed normal distributions, except items 9 (skewness = −2), 13 (skewness = 1.7), and 14 (skewness = −1.1). However, all of these items were retained. This is common with psychometric data, and principal axis factoring methods are shown to be robust to skewness levels beyond the parameters described above [17,18]. All items shared a Pearson’s correlation coefficient ≥ 0.3 with all other items except items 1 and 7. However, they were included, as individual communalities and the Kaiser–Meyer–Olkin (KMO) measures of sampling adequacy were ≥0.50 for all items [19] (Table 3 and Table 4). Therefore, all items were candidates for inclusion. The overall KMO measure was 0.80. Factors with an eigenvalue ≥ 1 were interpreted. A total of five factors were interpreted, which explained ~63% of the instrument’s variance. The factor correlation matrix is represented in Table 5. The factors identified are as follows: Professional Education (PE), Supplement Harm (SH), Professional Opinion (PO), Herbal Preparation (HP), and Dietitian Bias (DB).

**Figure 1:**
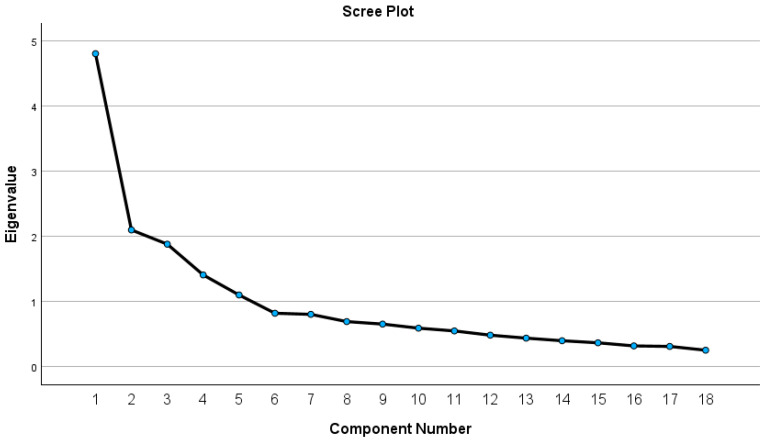
Scree plot.

The first factor, PE, explained 26.7% of the variance. It contained items 3, 4, 5, 6, 15, 16, 17, and 18. These items pertained to education and the recommendation of supplements within the dietitian’s scope of practice. The second factor, SH, explained about 11.7% of the variance. It contained items 8 and 11, which pertained to perceived harm and the ineffectiveness of dietary supplements. The third factor, PO, explained 10.5% of the variance and contained items 2, 9, and 12, which were items pertaining to the perceived support of including dietary supplement education during the training of the dietitian. The fourth factor contained items 13 and 14, which were items pertaining to feeling comfortable with using herbs in practice and was therefore named HP. The fifth factor contained items 1, 7, and 10, which appeared to be a Dietitian Bias construct which indicated questions that would address a more negative perception of how supplements were presented in training. The factors and their eigenvalues are shown in Table 4 and Figure 1.

PE, SH, and HP were reliable, with Cronbach’s α ≥ 0.70 (Table 5 and Table 6). However, PO and DB were not reliable according to this criterion. Item 7 was reverse-scored to calculate Cronbach’s alpha as it was the only item negatively correlated with the DB factor. PE shared weak to moderate negative relationships with PO and HP. The entire instrument was reliable (>0.70), regardless of the reverse scoring of item 7. Its reliability also remained consistent for any item deleted, further suggesting reliability in unity.

## 4. Discussion

In this study, we sought to validate an instrument that measured RDs/RDNs’ (in the United States) feelings of preparedness, comfort with use, and general attitudes in the field of dietary supplements. Questions were created and modified to establish face and content validity, with content validity being determined by exploratory factor analysis.

### 4.1. Factor Reliability

The guidelines used for interpreting Cronbach’s α reliability coefficient are as follows. A measurement of 0 indicates that all of the variance observed is due to measurement error, while α = 1 indicates only true score variance. The ratings of outstanding (α ≥ 0.9), very good (α ≥ 0.85), good (α ≥ 0.8), acceptable (α ≥ 0.75), borderline acceptable (α ≥ 0.7), okay (α ≥ 0.65), and unacceptable (α < 65) are assigned to reliability coefficients [16].

Based on these guidelines, PE, SH, and HP were reliable. PO demonstrated marginal unreliability, with DB being unreliable. As aforementioned, items 1 (“I think most people can get all their nutrition from food, without needing supplements.”) and 7 (“During my dietetics education, supplements were largely presented in a negative fashion.”) had correlations ≤ 0.3 with other items. However, they both had communalities and KMO scores of above 5, suggesting that they should be included. These items, along with item 10 (“I am knowledgeable discussing supplements from my dietetics training.”), were loaded onto DB (Dietitian Bias), which was unreliable (α ≥ 0.4). However, we recommend retaining DB and its items as they possess important clinical relevance. First, the questions passed face and content validity as assessed by 10 RDs/RDNs. In addition, these questions representing DB and PO may be lower in reliability because they are more general. However, these questions are purposely general because they represent opinions on average that are shaped from dietetic education that later form clinical beliefs and messaging to the general population. Another example of clinical relevance is that many programs do not offer much depth in providing counseling on specific supplement formulations for therapeutic use. For example, the information presented may include treating a person with iron deficiency anemia (a very common clinical situation) with iron supplements (most likely with vitamin C-rich foods), but it will not go into depth on what type of formulation should be used (such as iron sulfate vs. iron bisglycinate vs. sucrosomial iron vs. iron fumarate). These formulations will have different bioavailability and efficacy. Dietitians, with more experience and training specifically in dietary supplement formulations, should be more informed. We believe that further testing on larger populations will reveal these differences. We treated items 2, 9, and 12 (items that made up PO) similarly. Additionally, the entire instrument was reliable as a whole (α ≥ 0.7), with no substantial changes with any item removed (Table 6). This suggests that all items belong to the instrument in unity. PO and DB also shared significant moderate direct and strong inverse relationships (0.37 and −0.51) with the instrument. The factor loadings for PO and DB are also well above 0.5, suggesting strong relationships between the factors and their respective items. In summary, given the clinical/practical relevance, the relationships within the instrument, and reliability in the unity of the items in PO and DB, we chose to retain them. PE shared weak to moderate relationships with PO and HP. Item 7 was reverse-scored for the purpose of obtaining reliability. However, running the EFA both ways did not change the factor structure nor the amount of variance explained. Although DB and PO showed low reliability, their items were retained in the face and content validation stages.

### 4.2. The Scoring of the Instrument

The instrument may be scored by using the formula (1R + 2 + 3 + 4 + 5 + 6 + 7R + 8R + 9 + 10 + 11R + 12 + 13 + 14 + 15 + 16 + 17 + 18), where the numbers in the parentheses represent the respective items. Items 1R, 7R, 8R, and 11R are the reverse-scored items 1, 7, 8, and 11 respectively. These items represent negative attitudes toward supplements, while the other items represent positive attitudes and perceived self-efficacy in recommending supplements to patients. The total score ranges between 18 and 90.

The scores for all factors were found by summing their respective items, except for SH, which was found by summing 8R and 11R, and DB, which was found by summing items 1R, 7R, and 10. We may expect the factor structure to change in a population of non-American dietitians.

Investigators are encouraged to consider the nature of items 1, 7, 8, and 11 should the factor loadings differ in future studies to include the potential renaming of factors. Similarly to the entire instrument, we recommend using 1R, 7R, 8R, and 11R in computing scores for each factor.

It is difficult to compare the results to past studies because this is a new survey developed in an environment with different types of products. To our knowledge, there have been a handful of studies assessing uses and perceptions regarding dietary supplements among RDs/RDNs in the US [8,9,10,11,12,13], but there is no validated questionnaire assessing these constructs or questions on the feelings of preparedness on this topic. Prior studies also did not assess the feelings of comfort in dietetic practice based on training. From the results in Table 2, it is clear that there are some gaps that should be addressed in dietetic training. For example, while RDs/RDNs responded the most strongly that dietary supplements were within the scope of practice in nutrition (4.0 ± 0.8) and that they should understand this field to discuss it with patients (4.5 ± 0.7), they showed low scores in feeling prepared after their dietetic training (2.8 ± 1.1). Although this particular assessment was conducted to validate the questionnaire, larger representative studies are planned to assess the findings in more detail.

## 5. Conclusions

The objective of this study was to validate a questionnaire intended to address registered dietitians’ feelings of preparedness, comfort with use, and general attitudes in the field of dietary supplements. The questionnaire was shown to be valid and reliable in unity. Five factors were created: Professional Education, Supplement Harm, Dietitian Education, Herbal Preparation, and Dietitian Bias. Although Dietitian Education and Dietitian Bias were unreliable when analyzed as individual factors, these items were retained due to their clinical and educational relevancy since education during training can lead to clinical opinions and bias being brought into clinical practice, and the fact that the questionnaire’s overall internal consistency was acceptable was reliable in unity with all questions included. We recommend similar studies to be conducted in different countries with contrasting dietary education curricula with the expectation that the factor structure may differ. Although accredited programs should be uniform in training across the US, we still recommend that programs be evaluated for their coverage of availability and quality in terms of dietary supplement education and that the CDR consider more professional development for RDs/RDNs with CEUs since the field is continually evolving. We hope that in the future, the results of the questionnaire being used in larger populations of registered dietitians can serve as a foundation for additional training in dietetic curricula.

## Figures and Tables

**Table 1:** Demographic characteristics of responding registered dietitians (n = 222).

Characteristic	Result
**Age, average in years**	45.5 ± 13.7
**Years practicing dietetics, average in years**	17.2 ± 12.6
**Gender, n (%)**	
Female	204 (91.9)
Male	10 (4.5)
Gender diverse	6 (2.7)
Prefer not to answer	2 (0.9%)
**Highest level of education attained, n(%)**	
Bachelor’s degree	94 (24.3)
Master’s degree	120 (54.1)
Doctorate degree	8 (3.6)

**Table 2:** Questionnaire items and descriptive statistics.

	Questionnaire Item	Mean ± SD
1	I think most people can get all their nutrition from food, without needing supplements	3.4 ± 1.1
2	Supplements can be beneficial in improving health conditions	4.0 ± 0.7
3	I am comfortable evaluating evidence-based research on supplements for patient/clients	4.0 ± 0.8
4	I am comfortable recommending dosage to patients/clients	3.1 ± 1.1
5	I feel knowledgeable about bioavailable formulation of supplements (i.e., iron bisglycinate vs. sulfate)	2.6 ± 1.0
6	I am comfortable custom formulating supplements to meet the unique nutrient needs of my patients/clients.	2.4 ± 1.1
7	During my dietetics education, supplements were largely presented in a negative fashion.	3.1 ± 1.0
8	Many supplements can cause harm.	3.2 ± 0.9
9	RD/RDN’s should understand how and when to use supplements.	4.5 ± 0.7
10	I am knowledgeable discussing supplements from my dietetics training.	2.8 ± 1.1
11	Many supplements are ineffective	3.3 ± 1.0
12	College and university nutrition programs should provide more education on dietary supplements.	4.3 ± 0.7
13	I am comfortable with herbal preparations such as making syrups, decoctions, and infusions.	1.6 ± 0.9
14	I know how to use culinary herbs therapeutically (for example: to support respiratory symptoms)	1.9 ± 1.0
15	I feel well-prepared to discuss supplements with my clients	3.0 ± 1.0
16	I know where to find more education on dietary supplements	3.7 ± 1.1
17	Recommending supplements is within an RD/RDN’s scope of practice	4.0 ± 0.8
18	I am comfortable recommending supplements when a client is on prescription and/or over-the-counter medication	2.8 ± 1.1

**Table 3:** Questionnaire’s lower triangular correlation matrix (n = 222).

	1	2	3	4	5	6	7	8	9	10	11	12	13	14	15	16	17
**2**	-																
**3**	-	0.22 **															
**4**	-	0.21 **	0.49														
**5**	-	0.15 *	0.43	0.61													
**6**	−0.19 **	0.12 *	0.30	0.52	0.45												
**7**	−0.17 **	-	−0.11 *	-	-	-											
**8**	0.25	-	-	−0.14 *		−0.14	-										
**9**	-	0.46	0.34	0.22 **	0.16 **	0.13 *	-	0.13 *									
**10**	0.12 *	0.11	0.36	0.29	0.30	0.17 **	−0.26	−	0.12 *								
**11**	0.24	−0.18 **	-	−0.20 **	−0.14 *	−0.26	-	0.56	-	-							
**12**	-	0.28	-	-	-	-	0.13 *	-	0.36	−0.17 **	-						
**13**	-	-	-	0.16 *	0.16 **	0.29	-	-	-	-		-					
**14**	−0.13 *	-	0.17 **	0.26	0.30	0.28	-	−0.17 **	-	-	−0.18 **	0.14 *	0.63				
**15**	-	-	0.42	0.59	0.52	0.50	−0.14 *	-	0.14 *	0.38	−0.15 *	-	0.24	0.31			
**16**	-	-	0.49	0.50	0.41	0.26	-	-		0.34	-	-	0.18 **	0.28	0.47		
**17**	-	0.21 **	0.42	0.45	0.24	0.33	-	0.13 *	0.39	0.19 **	-	0.24	-	-	0.26	0.32	
**18**	−0.13 *	0.26	0.40	0.62	0.49	0.44	-	−0.15 *	0.18 **	0.25	−0.18 **	-	0.14 *	0.20 **	0.60	0.49	0.42

*p* < 0.001 unless otherwise indicated; * *p* < 0.05; ** *p* < 0.01; - indicate non-significant relationships.

**Table 4:** Questionnaire factor structure.

Item	Professional Education (PE)	Supplement Harm (SH)	Professional Opinion (PO)	Herbal Preparation (HP)	Dietitian Bias (DB)	KMO	Communalities
4	0.83					0.91	0.71
18	0.79					0.87	0.65
15	0.78					0.88	0.64
5	0.73					0.89	0.54
16	0.69					0.88	0.50
3	0.66					0.89	0.58
6	0.65					0.85	0.50
17	0.56					0.79	0.58
8		0.85				0.62	0.72
11		0.81				0.69	0.69
9			−0.80			0.72	0.67
12			−0.72			0.60	0.60
2			−0.71			0.71	0.59
13				0.89		0.59	0.80
14				0.88		0.65	0.82
7					−0.74	0.60	0.65
1					0.60	0.67	0.54
10					0.60	0.83	0.53
						**Totals**	
% variance	26.7	11.7	10.5	7.8	6.1	62.8	0.80
Eigenvalue	4.8	2.1	1.9	1.4	1.1		
Cronbach’s α	0.87	0.72	0.63	0.77	−	0.74	
Cronbach’s α (7R)					0.40	0.75	
Potential range	8–40	2–10	3–15	2–10	3–15	18–90	
Current range	9–40	2–10	3–15	2–10	3–12	30–77	
Mean ± S.D.	25.6 ± 5.8	5.5 ± 1.7	12.8 ± 1.6	3.5 ± 1.7	8.2 ± 1.8	55.6 ± 8.3	

KMO—Kaiser–Meyer–Olkin measure of sampling adequacy.

**Table 5:** Factor correlation matrix.

	PE	SH	PO	HP	DB
**SH**	−0.16 *				
**DE**	0.24 **	−			
**HP**	0.30 **	−0.16 *	−		
**DB**	−0.33 **	0.18 **	−	−	
**Q**	0.91 **	−0.4 **	0.37 **	0.47 **	−0.51 **

* *p* < 0.05; ** *p* < 0.01. Note: Correlation matrix was created using item summed scores and not principal axis factor scores.

**Table 6:** Internal consistency of questionnaire regarding dietary supplement attitudes and perceptions demonstrated by Cronbach’s alpha.

Q *	Cronbach’s α If Item Deleted	Cronbach’s α If Item Deleted (7R)
1	0.76	0.77
2	0.74	0.75
3	0.71	0.72
4	0.70	0.71
5	0.71	0.72
6	0.72	0.73
7	0.76	0.76
8	0.75	0.77
9	0.73	0.75
10	0.73	0.74
11	0.76	0.78
12	0.75	0.76
13	0.74	0.75
14	0.73	0.75
15	0.70	0.72
16	0.71	0.73
17	0.72	0.73
18	0.70	0.72

* Question item.

## Data Availability

The dataset is available upon request from the authors. The data are not publicly available due to the assurance of confidentiality in the consent form.

## Supplementary Materials

The following supporting information can be downloaded at: https://www.mdpi.com/article/10.3390/nu17152451/s1, The D-SCOPE Questionnaire (DOCS1); ChatGPT (GPT-4.0 [Large Language Model; Open AI; https://chat.openai.com/chat accessed on 24 July 2025) usage information.

## Abbreviations

The following abbreviations are used in this manuscript:

D-SCOPE	Dietitians’ Survey of Comfort, Opinions, and Experience in Supplements
RD/RDN	Registered Dietitian Nutritionist
KMO	Kaiser–Meyer–Olkin
PE	Professional Education
SH	Supplement Harm
PO	Professional Opinion
HP	Herbal Preparation
DB	Dietitian Bias
