# Supplementary material for: Validation of D-SCOPE Questionnaire: Dietitians’ Survey of Comfort, Opinions, and Perceptions on Education in Supplements
Source: Nutrients. 2025 Jul 28;17(15):2451. doi: 10.3390/nu17152451 (PMC12348582; doi:10.3390/nu17152451)
Supplement: Supplementary file 1 [file nutrients-17-02451-s001.zip › ChatGPT Supplementary Material.pdf]

ChatGPT footnote: ChatGPT (GPT-4.0 [Large Language Model; Open AI; https://chat.openai.com/chat) was prompted to recommend a few academic-style acronyms to be used to "Title a questionnaire designed for dietitians. This questionnaire addresses the perceptions around education, feelings of comfort giving advice, and attitudes around the field of dietary supplements". The following answers were received (we chose D-SCOPE):

- SPREAD: supplement perceptions, readiness, education attitudes of dietitians.
- D-SPARE: Dietitian's Survey on Perceptions, Attitudes, Readiness, and Education in Supplements.
- RECAP-DS: Readiness, Education, Comfort, Attitudes, and Perceptions on Dietary Supplements.
- CAPS-DQ: Comfort, Attitudes, and Perceptions Survey of Dietary Supplements among Dietitians Questionnaire.
- D-SCOPE: Dietitians' Survey of Comfort, Opinions, and Perceptions on Education in Supplements.
- DISCERN: Dietitians' Insights on Supplement Counseling, Education, Readiness, and Knowledge.
- PREDICT: Perceptions, Readiness, Education and Dietitians' Insight on Counseling about Therapeutics.
