# Supplementary material for: Validation of D-SCOPE Questionnaire: Dietitians’ Survey of Comfort, Opinions, and Perceptions on Education in Supplements
Source: Nutrients. 2025 Jul 28;17(15):2451. doi: 10.3390/nu17152451 (PMC12348582; doi:10.3390/nu17152451)
Supplement: Supplementary file 1 [file nutrients-17-02451-s001.zip › Supplement D scope Q.pdf]

The D-SCOPE Questionnaire (Dietitians' Survey of Comfort, Opinions, and Perceptions on Education in Supplements)

Directions: The following questions will ask you about your opinion about dietary supplements ***from your professional perspective. You do not need to be seeing clients to answer this survey.*** When answering the following questions regarding your **opinion** about dietary supplements, please consider the following to be a dietary supplement: Any ingredient intended to be added to the diet in any form (for example: teas, capsules, tablets, powders, tinctures) and include (but not limited to) vitamins, minerals, herbals, probiotics, prebiotics, enzymes, amino acids, fats (for example: MCT oil, borage oil, fish oil), glandular extracts, sport supplements (for example: creatine, whey protein), superfood extracts (for example: green powders, phytochemicals like quercetin, resveratrol).

Likert scale 1-5: (1- strongly disagree, 2-disagree, 3- neither agree nor disagree, 4-agree, 5-strongly agree)

	**1 Strongly Disagree**	**2 Disagree**	**3 Neither agree nor disagree**	**4 Agree**	**5 Strongly Agree**
1. I think most people can get all their nutrition from food, without needing supplements.					
2. Supplements can be beneficial in improving health conditions.					
3. I am comfortable evaluating evidence-based research on supplements for patients/clients.					
4. I am comfortable recommending dosage to patients/clients.					
5. I feel knowledgeable about bioavailable formulation of supplements (i.e., iron bisglycinate vs sulfate).					
6. I am comfortable custom formulating supplements to meet the unique nutrient needs of my patients/clients.					
7. During my dietetics education, supplements were largely presented in a negative fashion.					
8. Many supplements can cause harm.					

9. RD/RDN's should understand how and when to use supplements.					
10. I am knowledgeable discussing supplements from my dietetics training.					
11. Many supplements are ineffective.					
12. College and university nutrition programs should provide more education on dietary supplements.					
13. I am comfortable with herbal preparations such as making syrups, decoctions, and infusions.					
14. I know how to use culinary herbs therapeutically (for example: to support respiratory symptoms).					
15. I feel well-prepared to discuss supplements with my clients.					
16. I know where to find more education on dietary supplements.					
17. Recommending supplements is within an RD/RDN's scope of practice.					
18. I am comfortable recommending supplements when a client is on prescription and/or over-the-counter medication.					
